# Outcome of Thumb Reconstruction Using the First Dorsal Metacarpal Artery Island Flap

**Published:** 2018-05

**Authors:** Samir M Ghoraba, Wael H Mahmoud

**Affiliations:** Plastic, Reconstructive Surgery and Burns Department, Tanta Faculty of Medicine, Tanta, Egypt

**Keywords:** Thumb, Soft tissue, Defect, First dorsal metacarpal artery flap, Reconstruction

## Abstract

**BACKGROUND:**

Reconstruction of complex soft tissue defects of the thumb, with exposure of tendons, joints or bones, has always been a difficult task. We evaluated the functional and esthetic outcomes of 1^st^ dorsal metacarpal artery island flap in reconstruction of post-traumatic soft tissue defects of the thumb.

**METHODS:**

Between January 2012 and June 2014, fifteen patients with complex post-traumatic soft tissue thumb defects underwent 1^st^ dorsal metacarpal artery island flap. Sensory function was evaluated with static 2-point discrimination and cortical reorientation. The mobility of the thumb was tested by the Kapandji score. The esthetic outcome was assessed. Patient’s subjective satisfaction was evaluated by the visual analogue scale.

**RESULTS:**

The mean flap size was 33.3×17.7 mm. All donor sites were grafted by full-thickness skin grafts from the groin. Fourteen flaps survived completely and one had distal flap necrosis was treated conservatively. The mean static two-point discrimination was 10.4 mm. Cortical reorientation was complete in 40%. The average Kapandji score was 7.1. The esthetic outcome was excellent in six, good in eight and poor in one subject. After a mean follow up period of 18.2 months, the mean subjective satisfaction score was 8.1; most patients regained all functions of the thumb and index finger and were pleased with the cosmetic appearance of the flap and donor site.

**CONCLUSION:**

First dorsal metacarpal artery flap offers a sensate, pliable and versatile coverage for small to moderate sized thumb defects. Moreover, it provides good functional and esthetic outcomes with minimal donor site morbidity.

## INTRODUCTION

The thumb is used in almost all human hand functions.^1^ Therefore, thumb injuries have much more significant impact on the normal daily life activities than do other digits injuries.^2^ Reconstruction of complex soft tissue defects of the thumb, with exposure of the underlying structures, is challenging to hand surgeons due to limited local soft tissue availability and the requirements for pliable, durable and sensate skin coverage to preserve the thumb function which is of paramount value to the usefulness of the hand.^3^

Conventionally, these defects can be resurfaced by Moberg advancement flap,^4^ sensate cross-finger flap,^5^ Littler’s neurovascular island flap,^6^ first dorsal metacarpal artery (FDMA) flap,^7^ reversed radial forearm flap,^8^ distant flaps^9^ and various free flaps from the 1^st^ and 2^nd^ toes and web space of the foot.^10^ The 1^st^ dorsal metacarpal artery (FDMA) flap was first reported by Hilgenfeldt in 1961 and Hollevich in 1963 as a peninsular flap with preservation of the skin over the pedicle.^11^ An island flap was demonstrated for the first time by Foucher and Braun in 1979, who described that a sensate skin island flap could be harvested from the dorsum of the index finger, based on the 1^st^ dorsal metacarpal artery and incorporated a sensory branch of the superficial radial nerve.^12^

The 1^st^ dorsal metacarpal artery, has been found to be a constant vessel, originating from the radial artery in the 1^st^ intermetacarpal space just proximal to the point where it dives between both heads of the 1^st^ dorsal interosseous muscle and distal to extensor pollicis longus tendon. The FDMA runs suprafascially over the fascial layer of the 1^st^ dorsal interosseous muscle in 57% of cases, while it takes a subfascial course in 43% of patients, then divides into ulnar branch to the index finger, intermediate branch to the 1^st^ web space and radial branch to the thumb.^13^ The ulnar branch runs distally between the shaft of the 2^nd^ metacarpal bone and the ulnar head of the 1^st^ dorsal interosseous muscle until reaching the MCP joint, where it forms a functionally important anastomoses with the branches of the 2^nd^ dorsal and palmer metacarpal arteries, then it ramifies into a number of small vessels that supply the dorsal aspect of the index proximal phalanx through a rich subdermal plexus. The FDMA has two venae comitantes that are in connection with large cutaneous superficial veins in the 1^st^ intermetacarpal space; represent the venous drainage of the flap.^14^

Being a sensate flap with a constant vascular anatomy, the FDMA flap has been successfully used for reconstruction of thumb soft tissue defects. However, over the past decades, there has been a great debate about the ideal soft tissue coverage for thumb defects. This study was undertaken to evaluate the functional and esthetic outcomes of “1st dorsal metacarpal artery island flap” in reconstruction of post-traumatic soft tissue defects of the thumb.

## MATERIALS AND METHODS

This prospective study was performed, between January 2012 and June 2014, in the Plastic Surgery Department, Tanta University Hospitals, on fifteen patients with complex post-traumatic soft tissue thumb defects (11 dorsal and 4 volar) that were covered with 1st dorsal metacarpal artery island flaps. The patients included 2 females and 13 males ranging in age from 15 to 49 years (mean, 34.8 years). Ten patients were operated as emergency cases, while 5 had delayed surgery due to skin necrosis after trauma. The dominant hand was affected in twelve (80%) patients. Informed consent was taken from all patients after detailed description of the procedure. Approval of the University Ethical Committee was obtained before commencing the study.

**Fig. 1:**
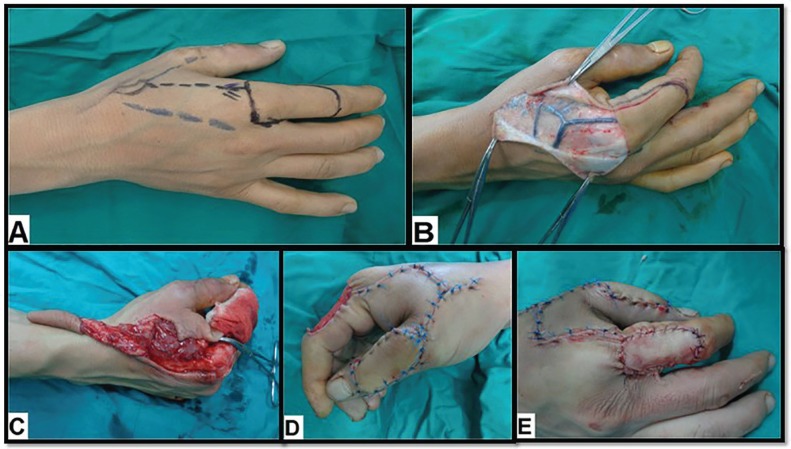
A 24-year old male with Rt post-traumatic dorsal thumb defect. **(A).** Pre-operative flap design. **(B).** Intra-operative view shows subdermal dissection to expose the pedicle. **(C).**Intra-operative view shows flap harvesting. **(D).** Intra-operative view shows flap in setting. **(E).** Intra-operative view shows grafting of the donor site by full-thickness skin graft

The flap was marked over the dorsal skin of the proximal phalanx of the index finger. The flap width was outlined so that it didn’t go beyond the ulnar and radial midaxial lines of the proximal phalanx and its margins distally and proximally were designed to maintain the dorsal skin of the proximal interphalangeal (PIP) joint and the metacarpophalangeal (MCP) joint respectively. Between the bases of the 1^st^ and 2^nd^ metacarpal bones, the radial artery pulsation was felt. The FDMA course was marked extending radial and parallel to the 2^nd^ metacarpal bone from the radial pulsation (Figure 1a). 

All patients were operated under general anaesthesia with the aid of pneumatic tourniquet control (250-300 mmHg) and loupe magnification. After debridement and preparation of the thumb defect, the flap was outlined according to the size of the defect. A lazy-S incision was done between the radial border of the metacarpophalangeal (MCP) joint and the tip of the triangular 1^st^ web space (the pivot point). Thereafter, subdermal dissection was done to expose the pedicle, with great care to avoid injury of the large superficial subcutaneous veins (Figure 1b). 

Elevation of the flap commenced from distal to proximal side and from ulnar to radial side preserving the paratenon to ensure the free gliding of the extensor tendon and the take of the skin graft. The pedicle was then dissected towards the pivot point. To ensure safe dissection, the extensor hood at the radial aspect of the metacarpophalangeal (MCP) joint, the periosteum of the radial shaft of the 2^nd^ metacarpal bone and the fascia of the ulnar head of the 1^st^ dorsal interosseous muscle must be included within the pedicle along with the sensory branch of the radial nerve and the dorsal veins. After flap harvesting, the tourniquet was released to ensure its vascularity (Figure 1c). 

The flap was then transferred through a subcutaneous tunnel into the defect of the thumb by gentle traction. The tunnel was tight in seven (46.7%) patients and had to be laid open then sutured primarily after flap in setting (Figure 1d). The donor sites were grafted by full-thickness skin grafts harvested from the groin in all patients (Figure 1e). After suturing and graft dressing were complete, a protective splinting was applied.

The hand and the fingers were immobilized in neutral position with dorsal splint for 10 days to ensure proper graft take. All patients were discharged after graft dressing on the 5^th^ post-operative day. Sutures were removed on 10-14^th^ post-operative day. This was followed by a course of physiotherapy for 6 weeks in all patients. The patients were instructed to come for post-operative follow up every month for 3 months, then every 3 months for three years. All patients were evaluated for the occurrence of early post-operative complications in terms of flap necrosis, hematoma, infection, wound dehiscence and graft loss. 

Sensory function was evaluated with static 2-point discrimination (s2-PD) testing. Cortical reorientation was tested by asking the patient whether the needle prick stimulation coming from the thumb or the index. The mobility of the 1^st^ ray was tested by thumb opposition using the Kapandji score^15^ (Table 1). The esthetic outcome was objectively assessed by 2 different plastic surgeons and was graded as poor, good and excellent. Patient’s subjective satisfaction was evaluated, regarding the functional recovery and the esthetic appearance of the flap and donor site, using the visual analogue scale (0=completely disappointed, 10=completely satisfied).

**Table 1:** **:** Kapandji score.^15^

**Score**	**Location achieved**
1	Radial side of the proximal phalanx of the index finger
2	Radial side of the middle phalanx of the index finger
3	Tip of the index finger
4	Tip of the middle finger
5	Tip of the ring finger
6	Tip of the little finger
7	Distal interphalangeal joint crease of the little finger
8	Proximal interphalangeal joint crease of the little finger
9	Metacarpophalangeal joint crease of the little finger
10	Distal palmar crease

## RESULTS

In two and half years, fifteen 1st dorsal metacarpal artery island flaps were used for reconstruction of complex post-traumatic soft tissue thumb defects in 15 cases with an average age of 34.8 years. The subjects’ data and outcomes are summarized in Table 2. The flap sizes ranged from 20×15 mm to 43×26 mm (mean 33.3×17.7 mm). Fourteen flaps survived completely and one had distal flap necrosis that was treated conservatively and healed by secondary intention. All the recipient and donor areas were healed uneventfully.

**Table 2:** Patient data and outcomes

**No.**	**Age (yr)/Sex**	**Defect site**	**Flap size (mm)**	**Wound complications**	**Functional outcome**			**Esthetic outcome**	**SS score**	**Follow-up (months)**
					**S2-PD (mm)**	**Cortical reorientation**	**Kapandji score**			
1	30/M	Lt. dorsal	38×23	No	14	Incomplete	7	Good	9	18
2	47/M	Rt. dorsal	29×18	No	6	Complete	8	Excellent	9	21
3	30/F	Rt. dorsal	35×16	No	15	Incomplete	5	Good	7	12
4	46/M	Rt. dorsal	32×20	No	13	Incomplete	7	Good	8	9
5	24/M	Rt. dorsal	43×26	No	7	Complete	9	Excellent	10	24
6	33/M	Lt. volar	30×15	No	12	Incomplete	8	Good	8	15
7	40/M	Rt. volar	36×18	Distal necrosis	10	Complete	7	Poor	4	30
8	15/M	Lt. dorsal	37×17	No	13	Incomplete	8	Good	7	18
9	28/F	Lt. dorsal	34×16	No	11	Incomplete	5	Excellent	8	12
10	45/M	Rt. volar	20×15	No	8	Complete	8	Excellent	9	21
11	36/M	Rt. dorsal	28×17	No	10	Incomplete	8	Good	9	15
12	49/M	Rt. dorsal	35×15	No	14	Incomplete	4	Good	6	12
13	26/M	Lt. dorsal	39×20	No	7	Complete	9	Excellent	10	30
14	34/M	Rt. volar	28×15	No	10	Incomplete	6	Good	8	15
15	39/M	Rt. dorsal	36×15	No	6	Complete	8	Excellent	10	21

M: Male; F: Female; Rt: Right; Lt: Left; s2-PD: Static two-point discrimination; SS score: Subjective satisfaction score

**Fig. 2:**
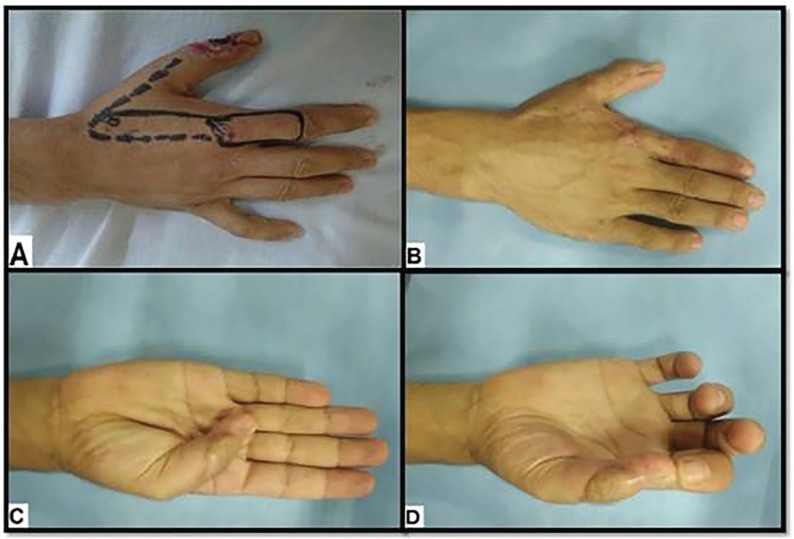
A 36-year old male with Rt post-traumatic dorsal thumb defect. **(A).** Pre-operative flap design. **(B).** Six months postoperative view shows good esthetic outcome**.** **(C, D).** Six months postoperative view shows good functional outcome

**Fig. 3:**
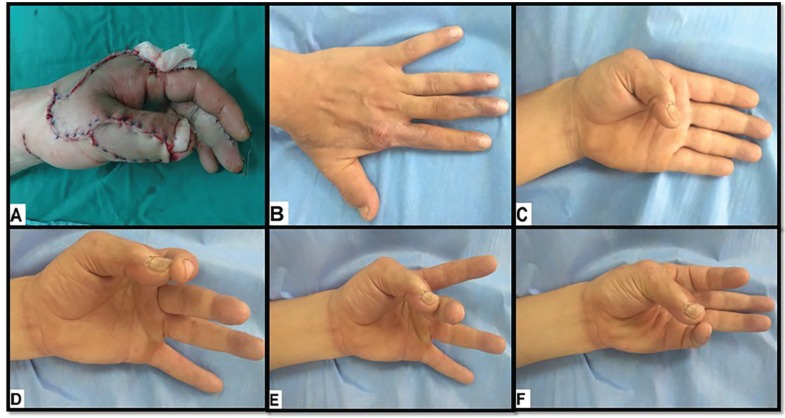
A 30-year old male with Lt post-traumatic dorsal thumb defect. **(A).** Intra-operative view. **(B).** Six months postoperative view shows good esthetic outcome**.** **(C, D, E, F).** Six months postoperative view shows good functional outcome

The mean follow-up period was 18.2 months (range 9-30 months). The static two-point discrimination (s2-PD) ranged from 6 to 15 mm; with an average of 10.4 mm. Cortical reorientation was complete in six (40%) patients. The average Kapandji score was 7.1 (range: 4-9). The esthetic outcome of the donor and recipient areas was excellent in six (40%) patients, good in eight (53.3%) patients and poor in one (6.7%) case. The mean subjective satisfaction (SS) score was 8.1 (range: 4-10); most patients regained all functions of the thumb and index finger and were pleased with the cosmetic appearance of the flap and donor site (Figure 2 and 3).

## DISCUSSION

Complex soft tissue defects of the thumb, with exposure of tendons, joints or bones have always been a challenging reconstructive task. Local, regional and free flaps have been used to resurface such defects. Nevertheless, each therapeutic modality has its own advantages and limitations. Local flaps^16^ offer superior esthetic results due to replacement “like with like” tissue. However, limited arc of rotation and limited amount of soft tissue availability are major drawbacks. The Moberg advancement flap^4^ is quite useful for thumb defects distal to the IP joint, but is not recommended for large ones due to risk of thumb IP flexion contracture. Although, the cross finger flap^5^ is frequently used as a salvage procedure, it is a staged one with the risk of finger joints stiffness and 1^st^ web contracture. 

Littler’s neurovascular island flap,^6^ harvested from the distal ulnar aspect of the middle or ring finger, is another therapeutic option. However, the major disadvantages of this flap are cold intolerance, poor cortical reorientation and loss of two-point discrimination. The reversed radial forearm flap^8^ is a versatile regional flap that could be used as a fasciocutaneous, adipofascial, or perforator based flap for reconstruction of nearly all thumb defects. However, donor site morbidity and sacrifice of one of the two major vascular axes of the forearm are the main limitations. Although distant flaps less commonly used, they remain a useful tool for complex soft tissue defects of the thumb.^17^

They are limited by the need for additional procedures for separation and debulking to optimize hand functions. Microvascular transfer of free flaps from the foot was described to remedy such problem. However, the needs for microsurgical experience and facilities, long operative time and moderate to poor sensory outcome are major handicaps.^10^ We aimed in this study to evaluate the functional and esthetic outcomes of “1st dorsal metacarpal artery island flap” in reconstruction of post-traumatic soft tissue defects of the thumb. The FDMA flap or Foucher’s flap is an island sensory flap based on the 1^st^ dorsal metacarpal artery and a branch of the superficial radial nerve. It was 1^st^ described by Foucher and Braun in 1979 to cover thumb defects.^12^

It also known as “kite flap” as the flap is raised with its neurovascular pedicle that resembles the kite. The 1^st^ dorsal metacarpal artery is quite reliable. In this series, we did not use Doppler study to trace its course. Contrary to us, Trankle *et al.*^18^ advocated the use of preoperative Doppler to mark the course of the FDMA. We observed that the FDMA has a constant anatomy which is consistent with de Rezedo *et al.*,^19^ who found that the 1^st^ and 2^nd^ dorsal metacarpal arteries are anatomically constant and they are very reliable as a source of pedicle flaps without the need for preoperative Doppler study.

In our study, the mean flap size was 33.3×17.7 mm and we had only one distal flap necrosis that was managed conservatively. In a similar study, Satish *et al.* used 9 FDMA flaps to cover post-traumatic thumb defects and found that the mean flap size was 33.3×19.4 mm and only one flap had partial necrosis that healed without 2^nd^ ry procedure.^20^ They reported that the size limitation is a drawback of this flap that couldn’t extend beyond the PIP distally. El-Khatib devised an extended version of the FDMA flap for reconstruction of combined palmar and dorsal thumb defects in five patients and observed complete survival of all flaps which could be attributed to presence of rich dermal-subdermal plexus, supplying the skin of the dorsal aspect of proximal and middle phalanges of the index finger, allowing harvesting the skin of the dorsal aspect of the middle phalanx as a random extension.^21^

In this study, we noticed that the mean value of static two-point discrimination (s2-PD) was 10.4 mm. Similarly, Ege *et al.*^22^ used 21 Foucher’s flaps for thumb reconstruction and had an average s2-PD of 10.8 mm. In another study, Chang *et al.* observed that the mean s2-PD was 8.1 mm. The ability of the brain to recognize any stimulation of the flap site as from the thumb not from the index is called “cortical reorientation”.^7^

In our series, we had complete cortical reorientation in six (40%) patients. The incomplete reorientation in the remaining patients didn’t interfere with their normal daily activities. We observed that the least period needed for reorientation was 21 months which is consistent with other studies,^23^^,^^24^ who reported that a two-year period is needed for complete reorientation. Trankle *et al.*^18^ studied the quality of sensation of innervated FDMA flaps in different age groups and found that 11 patients younger that fifty years had a s2-PD of 10.8 mm compared with 10.9 mm of 14 patients older than fifty years, complete cortical reorientation occurred in 5 patients younger than fifty years and in 7 patients older than fifty years and 22 (88%) cases were satisfied with the outcome. They reported that no significant age-related differences were present in the surgical outcome of the FDMA flap.

We observed that after a mean follow up period of 18.2 months, the average Kapandji score in our patients was (7.1). Similar to our study and findings, Muyldermans *et al.*^23^ noticed that the average Kapandji score, after a mean follow up period of 15.4 months, was 7.43 and concluded that the FDMA flap is of choice in coverage of thumb defect at the proximal phalanx and proximal segment of the distal phalanx. Our study showed that the mean subjective satisfaction score was 8.1 and most of our patients were satisfied with the functional and esthetic results. 

Our data confirmed Kola *et al.*,^25^ who found that the mean subjective satisfaction score was (9.3). They are also agreed Eski *et al.*,^26^ who used 14 FDMA flaps to correct post-burn thumb deformities and observed that all deformities were corrected with satisfactory cosmetic results and functional recovery and minimal donor site morbidity. Contrary to our results, Ege *et al.*^22^ noticed that the imperfect esthetic results are major limitations of this technique especially in females.

Ratcliffe *et al.*^27^ described the use of the FDMA flap to manage thumb defects in 5 patients and reported no morbidity related to the donor site. Cil *et al.*^28^ denied the donor site morbidity of FDMA flap in their study. Sherif^29^ described the use of 23 FDMA flaps for reconstruction of the 1^st^ web space and coverage of thumb dorsal surface, thumb palmar surface and hand dorsal surface defects. They reported complete flap survival and satisfactory functional and esthetic results. 

However, we recommend for a longer follow up period on a larger group of patients for better evaluation of the esthetic and functional outcomes of the FDMA flap. We can conclude that the 1^st^ dorsal metacarpal artery flap offers a sensate, pliable and versatile coverage for small to moderate sized defects of both dorsal and volar aspects of the thumb. Furthermore, it provides good functional and esthetic outcomes with minimal donor site morbidity. 

## CONFLICT OF INTEREST

None declared.

## References

[1] Ray E, Sherman R, Stevanovic M (2009). Immediate reconstruction of a non replantable thumb amputation by great toe transfer. Plast Reconstr Surg.

[2] Lai CH, Lai CS, Huang SH, Lin SD, Chang KP (2010). Free medial plantar artery perforator flaps for resurfacing of thumb defects. Ann Plast Surg.

[3] Prabhu M, Powar R, Sulhyan SR (2013). FDMA flap: a versatile technique to reconstruct the thumb. Int J Pharm Med & Bio Sc.

[4] Rehim SA, Chung KC (2014). Local flaps of the hand. Hand Clin.

[5] Woon CY, Lee JY, Teoh LC (2008). Resurfacing hemipulp losses of the thumb: The cross finger flap revisited: Indications, technical refinements, outcomes and long-term neurosensory recovery. Ann Plast Sur.

[6] Xarchas KC, Tilkeridis KE, Pelekas SI, Kazakos KJ, Kakagia DD, Verettas DA (2008). Littler’s flap revisited: An anatomic study, literature review, and clinical experience in the reconstruction of large thumb pulp defects. Med Sci Monit.

[7] Chang SC, Chen SL, Chen TM, Chuang CJ, Cheng TY, Wang HJ (2004). Sensate first dorsal metacarpal artery flap for resurfacing extensive pulp defects of the thumb. Ann Plast Surg.

[8] Mahmoud WH (2015). Radial Forearm Flap versus Radial Adipofascial Perforator Based Flap for Reconstruction of Hand Soft Tissue Defects. Donn J Med Med Sci.

[9] Ali A, Farag M, Safe K (2007). Reconstruction of Hand and Forearm Defects by Abdominal Thin Skin Flaps. Egypt J Plast Reconstr Surg.

[10] Adani R, Cardon LJ, Castagnetti C, Pinelli M (1999). Distal thumb reconstruction using a mini wrap-around flap from the great toe. J Hand Surg.

[11] Holevich J (1963). A new method of restoring sensibility to the thumb. J Bone Joint Surg.

[12] Foucher G, Braun JB (1979). A new island flap transfer from the dorsum of the index to teh thumb. Plast Reconstr Surg.

[13] Earley MJ (1986). The arterial supply of the thumb, first web and index finger and its surgical application. J Hand Surg.

[14] Sherif MM (1994). First dorsal metacarpal artery flap in hand reconstruction, I: anatomy study. J Hand Surg.

[15] Kapandji A (1986). Clinical test of apposition and counter-apposition of the thumb. Ann Chir Main.

[16] Hurren J, Cormack G (2000). The application of the rotation flap to the dorsum of the hand. Br J Plast Surg.

[17] Biswas D, Wysocki RW, Fernandez JJ, Cohen MS (2014). Local and regional flaps for hand coverage. J Hand Surg Am.

[18] Tränkle M, Sauerbier M, Heitmann C, Germann G (2003). Restoration of thumb sensibility with the innervated first dorsal metacarpal artery island flap. J Hand Surg.

[19] de Rezende MR, Mattar Júnior R, Cho AB, Hasegawa OH, Ribak S (2004). Anatomic study of the dorsal arterial system of the hand. Rev Hosp Clin Fac Med Sao Paulo.

[20] Satish C, Nema S (2009). First dorsal metacarpal artery islanded flap: A useful flap for reconstruction of thumb pulp defects. Indian J Plast Surg.

[21] El Khatib HA (1998). Clinical experiences with the extended first dorsal metacarpal artery island flap for thumb reconstruction. J Hand Surg [Am].

[22] Ege A, Tuncay I, Ercetin O (2002). Foucher’s first dorsal metacarpal artery flap for thumb reconstruction: evaluation of 21 cases. Isr Med Assoc J.

[23] Muyldermans T, Hierner R (2009). First dorsal metacarpal artery flap for thumb reconstruction: a retrospective clinical study. Strategies Trauma Limb Reconstr.

[24] Delikonstantinou IP, Gravvanis AI, Dimitriou V, Zogogiannis I, Douma A, Tsoutsos DA (2011). Foucher first dorsal metacarpal artery flap versus littler heterodigital neurovascular flap in resurfacing thumb pulp loss defects. Ann Plast Surg.

[25] Kola N (2016). Thumb Reconstruction Using Foucher’s Flap. J Med Sci.

[26] Eski M, Nisanci M, Sengezer M (2007). Correction of thumb deformities after burn: Versatility of first dorsal metacarpal artery flap. Burns.

[27] Ratcliffe RJ, Regan PJ, Scerri GV (1992). First dorsal metacarpal artery flap cover for extensive pulp defects in the normal length thumb. Br J Plast Surg.

[28] Cil Y, Eski M, Isik S (2008). First dorsal metacarpal artery adipofascial flap for thenar burn contracture releasing. Burns.

[29] Sherif MM (1994). First dorsal metacarpal artery flap in hand reconstruction II Clinical application. J Hand Surg.

